# Role of MRI as first-line modality in the detection of previously undiagnosed otosclerosis: a single tertiary institute experience

**DOI:** 10.1186/s13244-020-00878-3

**Published:** 2020-05-19

**Authors:** Bela Purohit, Katya Op de beeck, Robert Hermans

**Affiliations:** 1 Department of Radiology, University Hospitals, KU Leuven, Leuven, Belgium; 2 Department of Neuroradiology, National Neuroscience Institute, Singapore, Singapore

**Keywords:** MRI temporal bones, HRCT temporal bones, Otosclerosis, Perilabyrinthine/pericochlear enhancement

## Abstract

**Background:**

Otosclerosis causes conductive, sensorineural and mixed hearing loss (CHL, SNHL, MHL) and tinnitus in young adults. It is best diagnosed on high-resolution CT (HRCT). Occasionally, patients presenting with SNHL and/or tinnitus may undergo temporal bone MRI as the first investigation. In this study, we have described the role of MRI as the first-line modality in the detection of previously undiagnosed otosclerosis.

Using search words ‘MRI otosclerosis’ we found 15 cases in the PACS of our institute, (University Hospitals, KU Leuven, Belgium) from 2003 to 2018. Of these, 2 were known cases of otosclerosis, hence excluded from the study. The remaining 13 patients underwent MRI as first-line investigation for unilateral SNHL (8/13), bilateral SNHL (3/13), unilateral MHL (1/13) and bilateral pulsatile tinnitus (1/13). All MRI studies were reported by the same senior radiologist.

**Results:**

Of these 13 cases, 12 were reported as showing MRI features suspicious for otosclerosis. The typical positive findings in these cases were intermediate T1 signal and post-contrast enhancement in the perilabyrinthine/pericochlear regions. Out of 13 patients, 9 underwent subsequent HRCT, confirming otosclerosis in all. The single MRI which was reported as normal initially showed otosclerosis on HRCT. Retrospective evaluation of this MRI study showed subtle positive findings of otosclerosis.

**Conclusion:**

The end point of this study was to validate the subtle findings of otosclerosis on MRI, by comparison to the gold-standard modality HRCT. Our hypothesis is that in the appropriate clinical setting, familiarity with MRI features of otosclerosis would increase the diagnostic ‘catch’ in the first ‘net’ itself i.e. first-line MRI.

## Key points

- Patients presenting with classic clinical manifestations of otosclerosis like CHL/ MHL typically undergo HRCT which is considered the imaging modality of choice.
- Occasionally, patients with clinically undiagnosed otosclerosis presenting with SNHL or tinnitus may undergo primary MRI instead of HRCT.
- MRI findings of otosclerosis are subtle and may be overlooked, especially if MRI is performed as the first-line scan.
- Typical MRI features of otosclerosis include intermediate T1 signal and post-contrast enhancement in the perilabyrinthine and pericochlear regions.

## Background

Otosclerosis is a unique bony dysplasia of the otic capsule commonly seen in young and middle-aged adults. The disease is bilateral in almost 85% cases. Otosclerosis is characterised by the replacement of normal ivory-like enchondral bone by spongy vascular bone. This decalcified vascular bone of the otic capsule eventually recalcifies and becomes more solid. These phases of increased vascularity, decalcification and subsequent recalcification cause the typical clinical manifestations of this disease [1–3]. Two major patterns are seen: (1) fenestral type in which otosclerotic foci are restricted to the lateral wall of the otic capsule, especially around the oval window (OW) and round window (RW) and (2) retrofenestral or cochlear type, in which otosclerotic foci predominantly involve the pericochlear regions. Fenestral involvement may eventually progress to cochlear involvement; hence, these manifestations are considered as a continuum of the same disease process rather than separate entities. Fenestral otosclerosis typically causes stapes fixation and CHL. Cochlear otosclerosis may present with SNHL, MHL, vestibular symptoms and/or pulsatile tinnitus (PT) [1–4].

Patients presenting with the classic clinical manifestations of fenestral otosclerosis (CHL, absent stapedial reflexes) usually undergo non-contrast HRCT of the temporal bones which is the modality of choice for the diagnosis and grading of otosclerotic plaques [1–6]. However, occasionally, patients presenting with SNHL (who are not clinically suspected with otosclerosis) and/or PT may undergo MRI of the temporal bones as first-line imaging, instead of HRCT [7–9]. The MRI features of otosclerosis are not as well-known as HRCT features and this may lead to diagnostic difficulties when patients are imaged with MRI as the first line of investigation, especially with no clinical suspicion [1, 2, 7–9]. Literature review showed only a few case reports and small case series published about the role of MRI as the first-line modality in the diagnosis of otosclerosis [7–9]. The aim of our retrospective study was to describe and validate the subtle findings on temporal bone MRI, suggestive for the diagnosis of otosclerosis, by eventual comparison to the gold-standard examination for this disease, i.e. HRCT. To the best of our knowledge, ours is the largest case series of patients diagnosed with otosclerosis (in the absence of clinical suspicion) on primary MRI.

## Methods

This retrospective study was approved by the institutional (University Hospitals, KU Leuven, Belgium) ethics committee and need for informed consent was waived. Using key search words ‘MRI otosclerosis’, we found 15 cases in the PACS of the radiology department of our institute (University Hospitals, KU Leuven, Belgium), performed from 2003 to 2018. Of these 15 cases, 2 patients were known cases of otosclerosis, already treated with stapedectomy, and were excluded from the study. The remaining 13 patients had pre- and post-contrast MRI performed as first-line modality for the evaluation of unilateral SNHL to rule out acoustic neuroma (8 patients), heavy bilateral SNHL (3 patients), unilateral MHL (1 patient) and bilateral PT with unilateral MHL (1 patient). There was no clinical suspicion for otosclerosis in any of these 13 cases. All these MR studies were read and reported by the same senior consultant radiologist with more than 30 years of experience in head and neck radiology.

All 13 patients underwent first-line MRI on a 1.5 or 3 T scanner using a standard head coil, and comparable sequences on both machines. Detailed parameters used on the 3 T machine were as follows: axial T2W spin-echo sequence through the entire brain and posterior fossa (TR/TE 3000/80 ms, slice thickness 4 mm, FOV 230 mm, matrix 400 × 320 mm, acquired/reconstructed resolution 0.58 × 0.72 mm/0.45 × 0.45 mm), CISS sequence through the cerebellopontine angles and internal acoustic meati (IAM) (TR/TE 2000/180 ms, slice thickness 0.7 mm, FOV 160 mm, matrix 400 × 280, acquired/reconstructed resolution 0.4 × 0.57 mm/0.31 × 0.31 mm ) and axial T1W SE sequence through the IAM, repeated after injection of gadolinium in the axial and coronal plane (TR/TE 600/9.4 ms, slice thickness 2.2 mm, FOV 230 mm, matrix 320 × 240, acquired/reconstructed resolution 0.72 × 0.96 mm/0.48 × 0.48 mm). Subsequent HRCT of the temporal bones was performed on a multidetector CT scanner (depending on the machine, 110–120 kV, 100–250 mAs, slice thickness 0.4–0.5 mm, FOV 80 mm, in plane spatial resolution 0.15 × 0.15 mm), with reformattings in the axial and coronal planes, respectively in the plane of and perpendicular to the lateral semi-circular canals (SCC). All HRCT studies were read by same consultant radiologist.

## Results

Of these 13 cases, 12 cases were reported as showing MR imaging features suspicious for otosclerosis. One MRI was reported as normal. Subsequent HRCT was performed in 9 out of 13 patients and confirmed the findings of otosclerosis in all 9 cases. The single MR study which was reported as normal subsequently showed otosclerosis on HRCT. The correlation between primary MRI findings and subsequent HRCT findings in these 9 patients is described in Table 1.

**Table 1:** Clinical presentation, MRI and HRCT findings of 9 patients who underwent first-line MRI and ultimately confirmed as otosclerosis on subsequent HRCT

Case number/sex/age	Clinical/audiometric presentation	Preliminary MRI findings	Interval between MRI and HRCT	Subsequent HRCT findings	Correlation between MRI and HRCT findings
1/F/43	Asymmetric (R) SNHL	Intermediate T1 signal and PCE in (B/L) FA regions + pericochlear regions	1 month	B/L fenestral and pericochlear hypodense plaques	MRI findings closely match HRCT
2/M/28	Asymmetric (L) SNHL	Reported normal	4 months	B/L fenestral hypodense plaques. No pericochlear disease	Otosclerosis missed on MRI at first read. Retrospective evaluation showed very subtle focal PCE in (B/L) FA
3/F/57	Asymmetric (L) SNHL	Intermediate T1 signal and PCE in (B/L) FA + pericochlear regions. Also enhancement around (B/L) SCC	2 weeks	B/L fenestral and pericochlear hypodense plaques. Also extensive plaques around B/L SCC	MRI findings closely match HRCT
4/M/41	Asymmetric (L) SNHL	Intermediate T1 signal and PCE at bilateral FA + (R) pericochlear region	1 month	B/L fenestral and pericochlear hypodense plaques. (R) OW occluded, (L) OW narrowed	(L) pericochlear disease not detected on MRI
5/M/47	(B/L) heavy SNHL	Intermediate T1 signal and PCE in (B/L) FA + pericochlear regions	18 months	B/L fenestral and pericochlear hypodense plaques	MRI findings closely match HRCT
6/M/61	Asymmetric (L) SNHL	Intermediate T1 signal and PCE in (B/L) FA + pericochlear regions. Also T2 hyperintensity in (B/L) pericochlear regions	2 months	B/L fenestral and pericochlear hypodense plaques. (L) RW obliterated	MRI findings closely match HRCT
7/M/64	(B/L) heavy SNHL	Intermediate T1 signal and PCE in (B/L) FA + pericochlear regions. Also T2 hyperintensity in (B/L) pericochlear regions. Right IAC diverticulum	1 week	B/L fenestral and pericochlear hypodense plaques Right IAC diverticulum	MRI findings closely match HRCT
8/M/46	(R) MHL	Intermediate T1 signal and PCE in (B/L) FA + pericochlear regions	1 month	B/L fenestral and pericochlear hypodense plaques. (R) OW obliterated	MRI findings closely match HRCT
9/F/61	(B/L) heavy SNHL	Intermediate T1 signal and PCE in (B/L) FA + pericochlear regions. Possible tiny (R) IAC diverticulum	1 month	B/L fenestral and pericochlear hypodense plaques. Right IAC diverticulum	MRI findings closely match HRCT

(*R*) right, (*L*) left, (*U/L*) unilateral, (*B/L*) bilateral, *FA* fissula antefenestram, *OW* oval window, *RW* round window, *PCE* post-contrast enhancement, *IAC* internal auditory canal

**Fig. 1:**
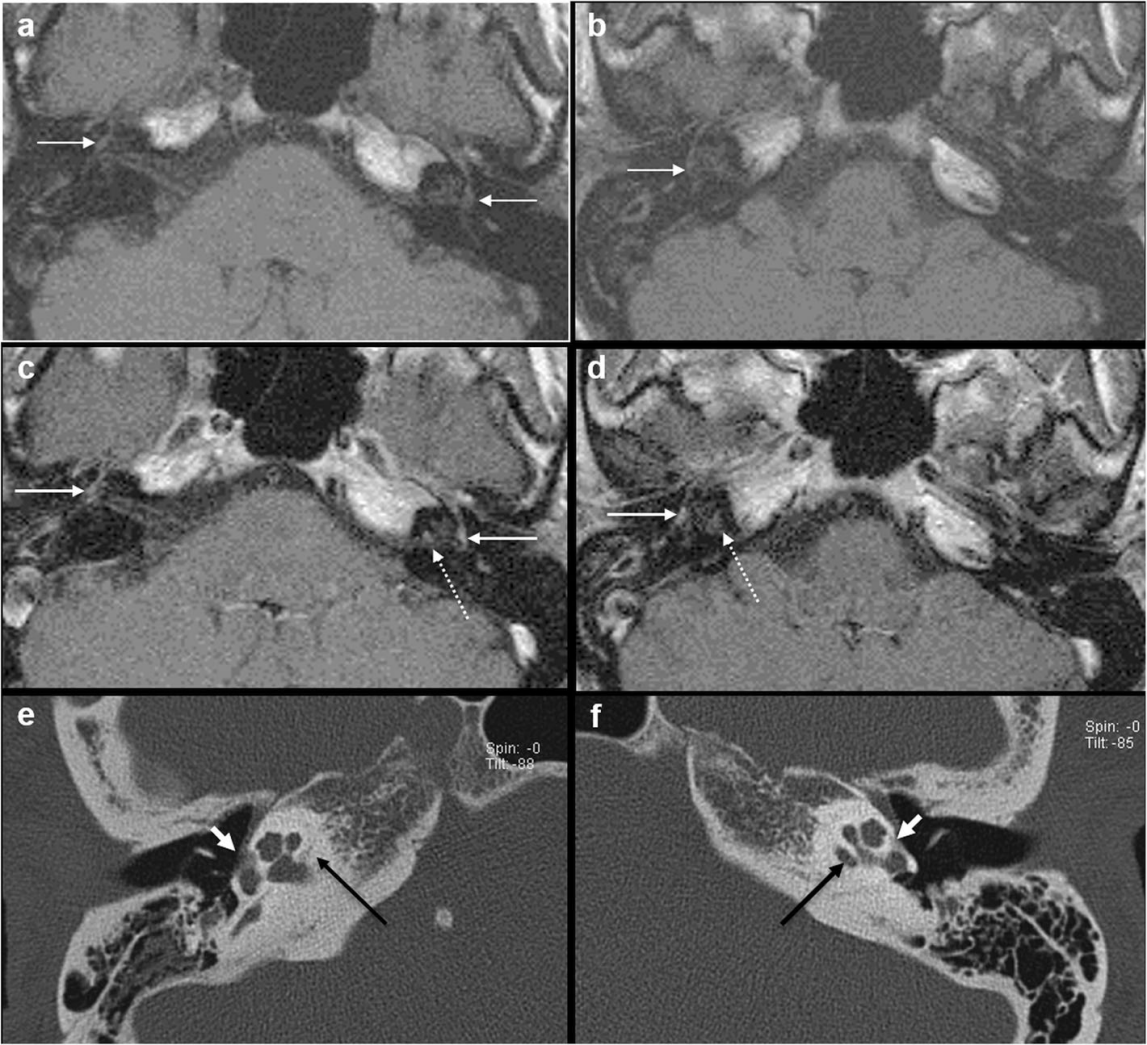
A 43-year-old female patient with right-sided SNHL (case 1). Axial T1W MR images (**a**, **b**) show subtle intermediate T1 signal in bilateral FA regions (arrows). Contrast-enhanced axial T1W images (**c**, **d**) show moderate post-contrast enhancement in bilateral FA regions (arrows) and mild post-contrast enhancement in bilateral pericochlear regions (dashed arrows). Subsequent axial HRCT images (**e**, **f**) confirm bilateral fenestral otosclerotic plaques (short white arrow) and pericochlear plaques (black long arrows)

**Fig. 2:**
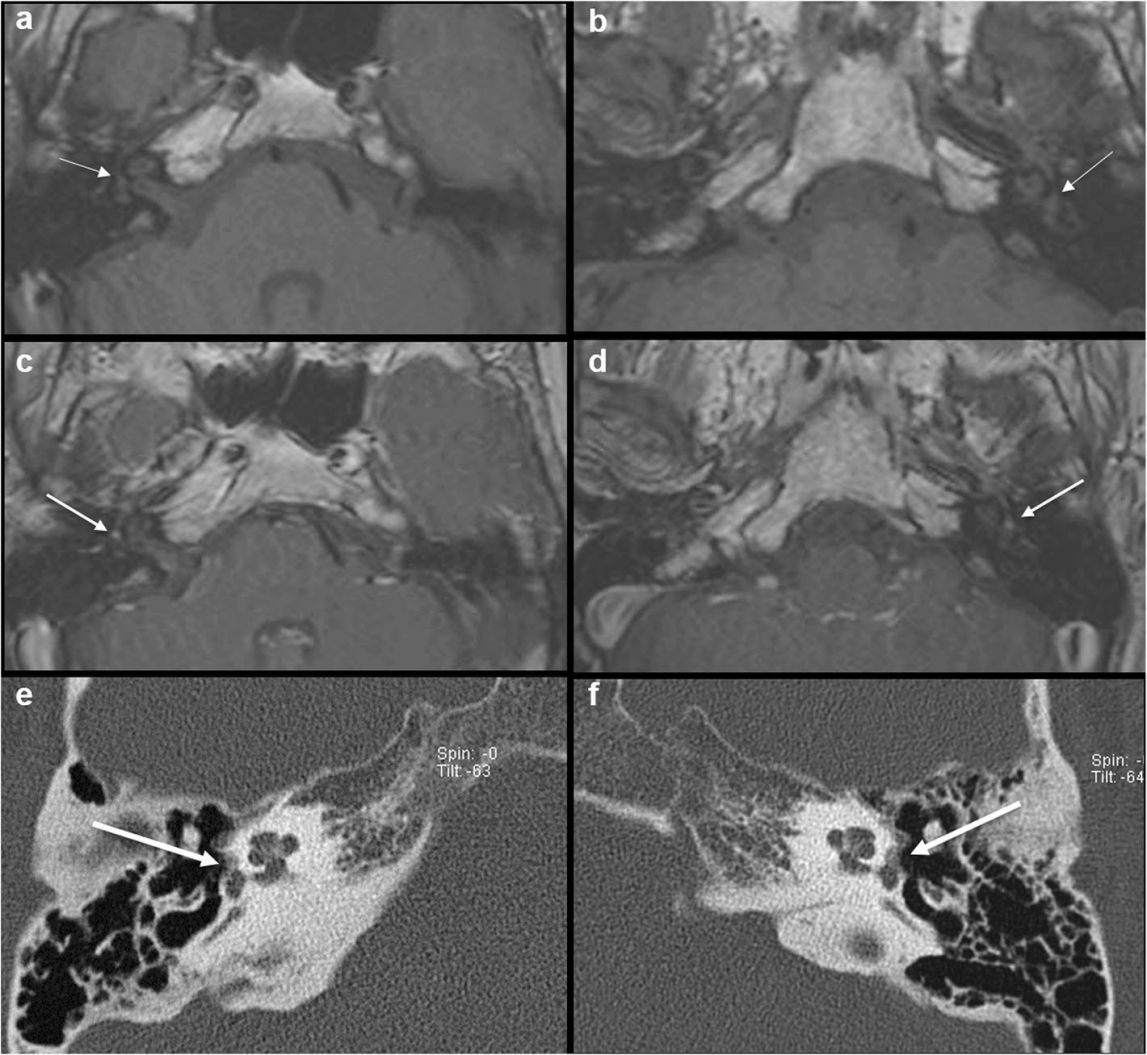
A 28-year-old male patient with left-sided SNHL (case 2). This was the case where otosclerosis was missed on first read MRI. Retrospective evaluation of axial T1W MR images (**a**, **b**) shows very subtle intermediate T1 signal in bilateral FA regions (arrows). Contrast-enhanced axial T1W images (**c**, **d**) show subtle focal post-contrast enhancement in bilateral FA regions (arrows). Axial HRCT images (**e**, **f**) show bilateral fenestral otosclerotic plaques (arrows). There was no pericochlear disease seen on HRCT

**Fig. 3:**
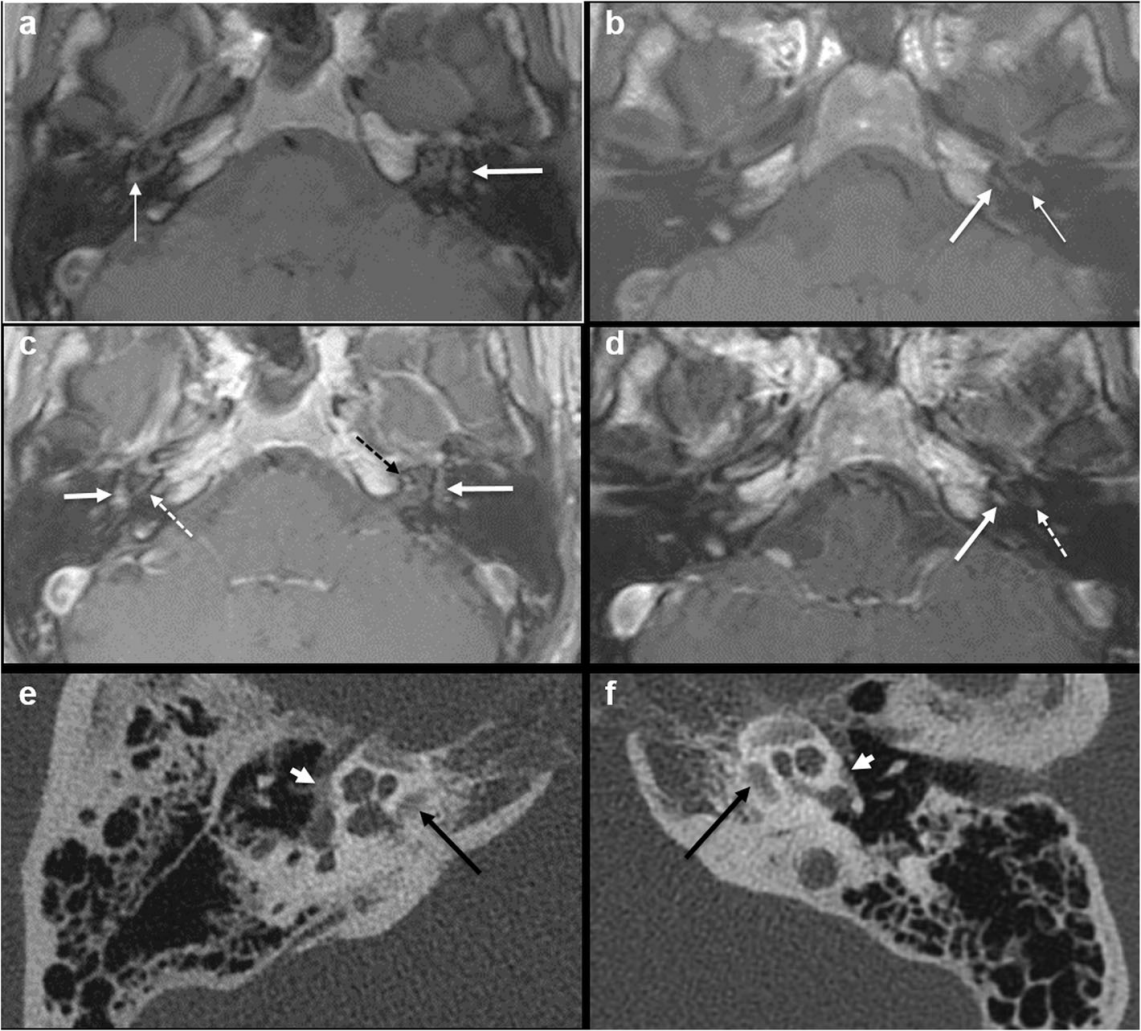
A 46-year-old male patient with right-sided MHL (case 8). Axial T1W MR image (**a**) shows intermediate T1 signal around the basal turn of the right cochlea (thin arrow) and in the left FA region (thick arrow). Axial T1W MR image at a lower level (**b**) shows intermediate T1 signal around the basal turn of the left cochlea (thin arrow) and another separate focus of intermediate signal in the left pericochlear region (thick arrow). Contrast-enhanced axial T1W image (**c**) shows moderate post-contrast enhancement in bilateral FA regions (arrows) and mild enhancement around the basal turns of cochleae (dashed arrows). Contrast-enhanced axial T1W image at a lower level (**d**) shows mild enhancement around the basal turn of left cochlea (dashed arrow) and another focus of enhancement in the left pericochlear region (arrow). Subsequent axial HRCT images (**e**, **f**) confirm bilateral fenestral otosclerotic plaques (short white arrows) and pericochlear plaques (black long arrows)

**Fig. 4:**
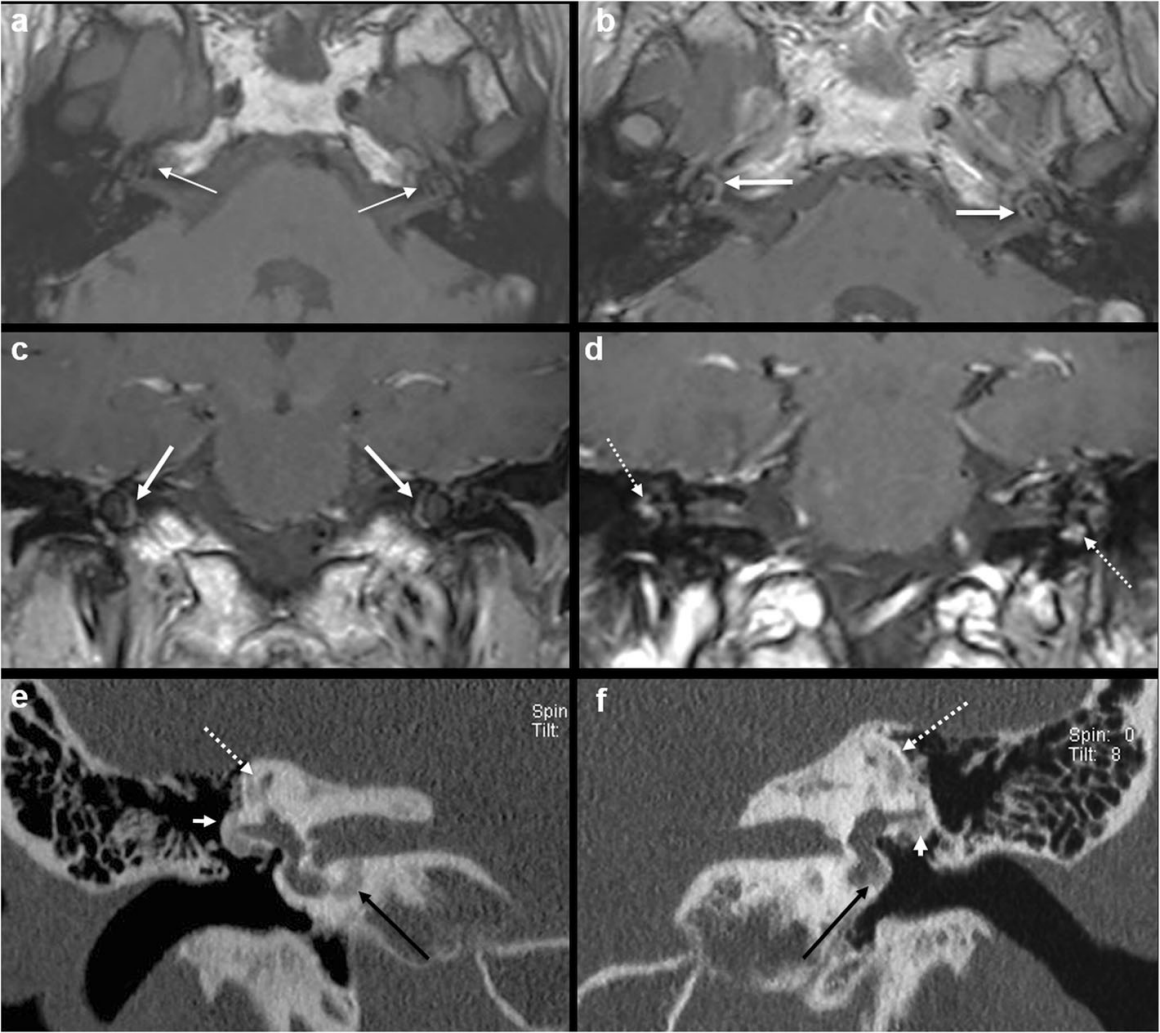
A 57-year-old female patient with left-sided SNHL (case 3). Axial T1W MR image (**a**) shows intermediate T1 signal in in bilateral pericochlear regions (arrows). Contrast-enhanced axial T1W MR image (**b**) and contrast-enhanced coronal T1W MR image (**c**) show ring-like enhancement in bilateral pericochlear regions (arrows). Contrast-enhanced coronal T1W MR image (**d**) also shows patchy enhancement surrounding the SCC bilaterally (dashed arrows). Subsequent coronal HRCT images (**e**, **f**) confirm otosclerotic plaques adjacent to bilateral lateral SCC (short white arrow), superior SCC (dashed arrow) and in bilateral pericochlear regions (long black arrows)

**Fig. 5:**
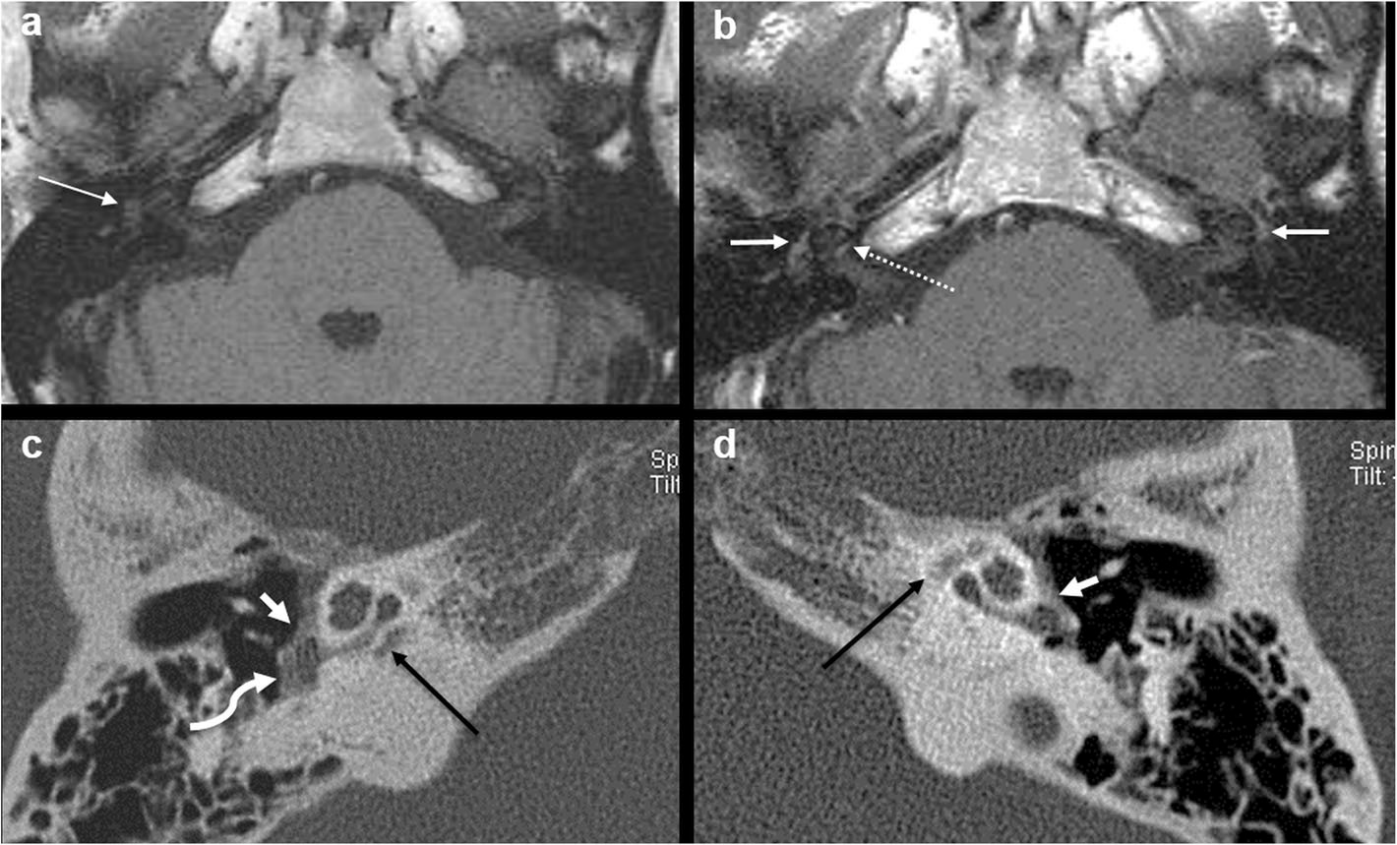
A 41-year-old male patient with left-sided SNHL (case 4). Axial T1W MR image (**a**) shows intermediate T1 signal in the right FA (arrow). Contrast-enhanced T1W MR image (**b**) shows moderate enhancement in bilateral FA regions (arrows) and mild enhancement in the right pericochlear region (dashed arrow). Pericochlear enhancement was not seen on the left. Subsequent axial HRCT images (**c**, **d**) confirm bilateral fenestral otosclerotic plaques (short white arrows) and pericochlear plaques (black long arrows). The right RW is occluded (curved arrow)

Intermediate T1 signal in bilateral fissulae antefenestram (FA) followed by mild-moderate post-contrast enhancement in these locations was seen in all 8 cases reported suspicious for otosclerosis on MRI (Fig. 1). The single MRI which was reported as normal initially (case no. 2) also showed these findings on retrospective evaluation, although they were very subtle (Fig. 2). In addition to these findings, intermediate T1 signal and mild-moderate post-contrast enhancement in bilateral pericochlear regions were seen in 7 out of the 8 positive cases (Figs. 3 and 4). Of these 7 cases, 1 case also showed enhancement on MR around bilateral SCC (case 3) (Fig. 4). All these 7 cases were reported as ‘suspicious for bilateral fenestral and cochlear otosclerosis on MRI, further confirmation with HRCT suggested’. One case showed pericochlear enhancement only on the right (case no. 4); this was reported as ‘suspicious for bilateral fenestral otosclerosis, also likely cochlear involvement on the right, further confirmation with HRCT suggested’ (Fig. 5). The single MRI which was reported as normal initially did not show pericochlear enhancement on either side on retrospective evaluation.

Subsequent HRCT showed bilateral fenestral and pericochlear hypodense plaques i.e. fenestral and cochlear otosclerosis in all 8 cases which were diagnosed as otosclerosis on MRI. The case which was reported as normal on MRI showed bilateral fenestral otosclerosis on HRCT.

**Fig. 6:**
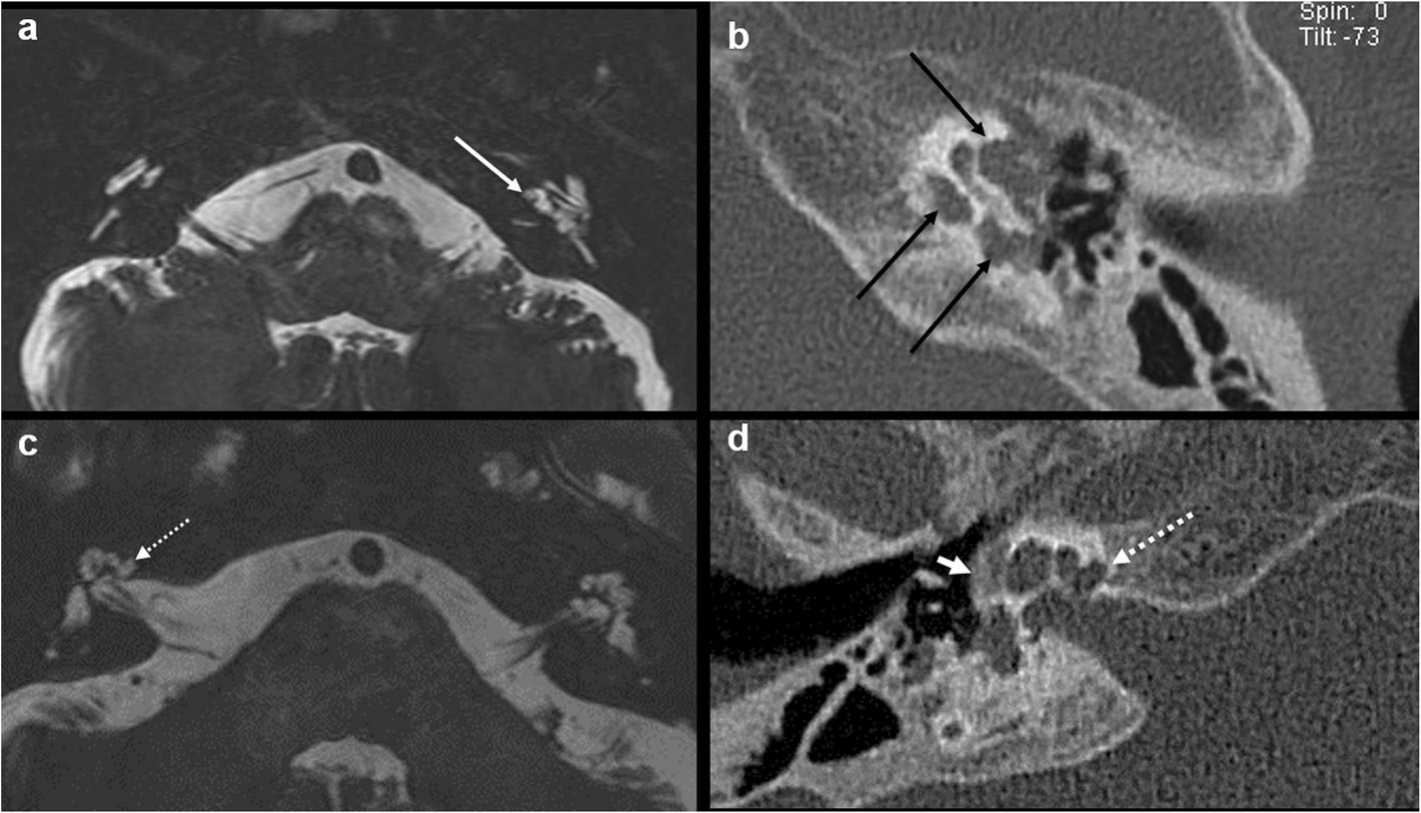
A 64-year-old male patient with heavy bilateral SNHL (case 7). Axial CISS image (**a**) shows curvilinear T2 hyperintensities (arrow) surrounding the left cochlea. Subsequent axial HRCT image (**b**) confirms extensive very hypodense plaques (arrows) in the left pericochlear region, likely long-standing disease. Axial CISS image (**c**) at a lower level also shows a small right IAC diverticulum (dashed arrow), confirmed on subsequent axial HRCT image (**d**) as an ‘ear-like’ outpouching anterior to the right IAC (dashed arrow). Also note the large plaque in the right FA region (short arrow)

Curvilinear non-enhancing T2 hyperintensities were seen in bilateral pericochlear regions in 2 out of the 7 cases with matching MRI and HRCT findings (case nos. 6 and 7) (Fig. 6); MRI detected IAC diverticula confirmed on HRCT in 2 out of the 7 matched cases (case nos. 7 and 9) (Fig. 6).

## Discussion

The initial lytic, active or ‘spongiotic’ phase of otosclerosis is characterised by increased cellularity, increased vascularity and decalcification of the otic capsule. The later phase, i.e. the classic ‘sclerotic’ phase is characterised by decreased cellularity, obliteration of blood vessels and formation of dense lamellar bone in these lesions [1–4, 7–9]. In fenestral otosclerosis, patients typically experience CHL due to otosclerotic plaques involving and eventually obliterating the margins of the OW and the footplate of the stapes. Cochlear otosclerosis is usually a continuum of fenestral otosclerosis, where foci of demineralised spongy vascular bone are seen in the cochlear capsule, sometimes around the IAC, rarely also around the SCC. Direct injury to the cochlea and spiral ligament due to the lytic/proteolytic enzymes, hyalinisation of the spiral ligament and vascular shunt-related hypoxia have been implicated as likely causes of the SNHL occurring in this phase. Vestibular symptoms are known to occur due to vestibular/labyrinthine involvement [1–4, 7, 8]; also, PT may occur due to increased vascularity/arteriovenous microfistulae in the otic capsule [1, 2, 10, 11]. Sometimes, patients with fenestral and cochlear otosclerosis may present with MHL due to a combination of CHL at the OW and SNHL at the cochlea [1–4, 8].

The clinical diagnosis of fenestral otosclerosis is based on the classic findings of CHL/Carhart’s notch (50–60 dB), absent stapedial reflexes, clean middle ear cavity and normal tympanic membrane. These patients typically undergo HRCT of the temporal bones as first-line imaging. HRCT plays an important role in detecting the size and location of otosclerotic plaques, assessing the status of OW and RW and ruling out concurrent middle ear/inner ear pathology [1–6, 8]. HRCT can detect very small hypodense demineralised otosclerotic foci, first occurring at the region of the FA, anterior to the OW. As the disease progresses, hypodense plaques are seen to extensively involve the margins of the OW, RW and eventually, the pericochlear regions. The classic HRCT imaging features of cochlear otosclerosis are distinctive bilateral pericochlear hypodense rings (described in old literature as the ‘4th ring of Valvassori’) [1–6]. Small IAC diverticula (focal hypodense outpouchings at the anterolateral IAC) are also described as HRCT features of advanced/cavitatory otosclerosis [12, 13]. Hypodense haloes may also be seen around the SCC in advanced disease [1, 2].

Occasionally, patients with otosclerosis who present with unilateral/asymmetric SNHL and /or vestibular symptoms are clinically suspected with retrocochlear pathologies such as acoustic neuroma. Also, unilateral/bilateral PT may be clinically confounding [1, 2, 7–11]. These patients may undergo a primary MRI of the temporal bones instead of HRCT [7–9, 14]. The MRI features of otosclerosis are often very subtle and radiologists may be unfamiliar with them [7–9, 14–19]; this may lead to potential misdiagnosis, especially in the absence of clinical suspicion [7–9].

Literature review (Medline search from 1990 to 2019) shows 3 case reports/case series describing the role of MRI as the primary modality in diagnosing clinically unsuspected otosclerosis [7–9]. Youssef et al. [7] describe a single case of a 48-year-old lady with clinical findings of bilateral SNHL, vertigo and unilateral tinnitus who underwent temporal bone MRI to rule out a retrocochlear pathology. MRI showed soft tissue signal and post-contrast enhancement in bilateral perilabyrinthine and pericochlear regions; it was reported as suspicious for otosclerosis and confirmed on subsequent HRCT. Goh et al., in their case series [8], have described 4 adult cases (39, 50, 45 and 55 years old) with bilateral MHL, unilateral SNHL, unilateral tinnitus and bilateral MHL, and bilateral SNHL respectively. All 4 cases underwent temporal bone MRI as first-line imaging. None of the cases were clinically suspected with otosclerosis. The positive findings in these cases leading to suggestion of otosclerosis were intermediate T1 signal in the perilabyrinthine and pericochlear regions, mild-moderate enhancement in these regions on post-contrast images and in two cases, pericochlear T2 hyperintense signal. Subsequent HRCT confirmed fenestral and cochlear otosclerosis in all 4 cases. Stimmer et al. described a single case of a 29-year-old lady who underwent primary temporal bone MRI to evaluate her bilateral MHL. MRI showed increased T1 and T2 signal with strong post-contrast enhancement in the perilabyrinthine regions. HRCT confirmed bilateral fenestral and cochlear otosclerosis.

A few other authors have also described similar MRI features of otosclerosis [14–19]. However, in all these studies, otosclerosis was either already known over years [17] or primarily diagnosed with a positive initial HRCT and MRI was performed as second-line imaging [14–16, 18, 19].

To the best of our knowledge, our study describes the largest number of patients from a single institute (8 cases) correctly diagnosed with otosclerosis, in the absence of clinical suspicion, on primary MRI, prior to HRCT. In our experience too, the imaging findings of intermediate T1 signal and mild-moderate post-contrast enhancement in the perilabyrinthine and pericochlear regions were most crucial for suspecting otosclerosis (see Table 1). We detected extensive enhancement around bilateral SCC in 1 of our positive cases. As described by Goh et al. and Ziyeh et al., we also detected pericochlear curvilinear non-enhancing T2 hyperintensities in 2 of our positive cases; these regions appeared as very dark/very low-attenuation haloes on subsequent HRCT, presumably indicating chronic disease [8, 15]. Interestingly, we also detected tiny unilateral IAC diverticula in 2 of the positive cases on MRI, confirmed on subsequent HRCT.

In summary, in 7 out of our 9 cases, MRI findings of bilateral fenestral and cochlear otosclerosis matched perfectly with those of subsequent HRCT. Of the remaining 2 cases, MRI correctly detected bilateral fenestral otosclerosis and unilateral cochlear involvement in 1 case; otosclerosis was missed entirely on MRI in 1 case. This case series shows a very good ‘positive catch of otosclerosis’, in the first ‘diagnostic net’ itself i.e. MRI, even in the absence of clinical suspicion. This correct diagnosis prompted the clinician to obtain the appropriate confirmatory diagnostic test, i.e. HRCT, thus leading to adequate clinical management. Our hypothesis is that in the appropriate clinical setting (young or middle-aged patients with SNHL, MHL and/or tinnitus), familiarity with MRI features of otosclerosis and a high index of suspicion would aid the reporting radiologist in arriving at the correct diagnosis.

Some of the limitations of our study include the relatively small data set from a single institute and the fact that all the MRI scans as well as the subsequent HRCT studies were read by the same senior experienced head-neck radiologist. Moving forward, it would be interesting to have more studies performed and published on this subject, with larger number of cases and comparison between various readers for assessing pick-up rate/variability in pick-up of subtle MRI findings of otosclerosis.

The MRI features of cochlear otosclerosis may be mimicked by other diseases that demineralise the otic capsule, typically osteogenesis imperfecta and Paget’s disease. These conditions also show soft tissue signal on T1 and post-contrast enhancement in the otic capsule. The HRCT findings may also be very similar, especially in otosclerosis and osteogenesis imperfecta. However, the entirely different spectrum of clinical manifestations and involvement of other bones are sufficient to differentiate between these pathologies [1–3, 8, 20–22].

Fenestral otosclerosis is treated by stapedotomy and a stapes prosthesis insertion. Cochlear otosclerosis on the other hand may be medically treated with fluorides. Patients with profound bilateral SNHL due to otosclerois may derive benefit from cochlear implantation [1–4, 8].

## Conclusion

The MRI findings of otosclerosis are subtle and may be overlooked. Keeping in mind the clinical context and being conversant with these imaging features is likely to increase the pick-up of these cases on MRI. By recommending the correct subsequent investigation i.e. HRCT, further diagnostic delay can be avoided and the correct treatment installed.

## Data Availability

Approved from institutional committee

## Abbreviations

CEMRI: Contrast-enhanced MRI
CHL: Conductive hearing loss
FA: Fissula antefenestram
HRCT: High-resolution CT
IAC: Internal auditory canal
IAM: Internal acoustic meatus
MHL: Mixed hearing loss
OW: Oval window
PT: Pulsatile tinnitus
RW: Round window
SCC: Superior semi-circular canal
SE: Spin echo
SNHL: Sensorineural hearing loss
